# Experimental validation of rotating detonation for rocket propulsion

**DOI:** 10.1038/s41598-023-40156-y

**Published:** 2023-08-30

**Authors:** John W. Bennewitz, Jason R. Burr, Blaine R. Bigler, Robert F. Burke, Aaron Lemcherfi, Tyler Mundt, Taha Rezzag, Ethan W. Plaehn, Jonathan Sosa, Ian V. Walters, S. Alexander Schumaker, Kareem A. Ahmed, Carson D. Slabaugh, Carl Knowlen, William A. Hargus

**Affiliations:** 1 Rocket Combustion Devices Branch, Air Force Research Laboratory, Edwards AFB, CA 93524 USA; 2 Jacobs Technology Group, Edwards, CA 93524 USA; 3 Propulsion and Energy Research Laboratory, University of Central Florida, Orlando, FL 32816 USA; 4 Maurice J. Zucrow Laboratories, Purdue University, West Lafayette, IN 47907 USA; 5 William E. Boeing Department of Aeronautics and Astronautics, University of Washington, Seattle, WA 98195 USA; 6 Naval Research Laboratory, Washington, DC 20375 USA; 7 Turbine Combustion Devices Branch, Air Force Research Laboratory, Wright-Patterson AFB, OH 45433 USA

**Keywords:** Aerospace engineering, Energy science and technology

## Abstract

Space travel requires high-powered, efficient rocket propulsion systems for controllable launch vehicles and safe planetary entry. Interplanetary travel will rely on energy-dense propellants to produce thrust via combustion as the heat generation process to convert chemical to thermal energy. In propulsion devices, combustion can occur through deflagration or detonation, each having vastly different characteristics. Deflagration is subsonic burning at effectively constant pressure and is the main means of thermal energy generation in modern rockets. Alternatively, detonation is a supersonic combustion-driven shock offering several advantages. Detonations entail compact heat release zones at elevated local pressure and temperature. Specifically, rotating detonation rocket engines (RDREs) use detonation as the primary means of energy conversion, producing more useful available work compared to equivalent deflagration-based devices; detonation-based combustion is poised to radically improve rocket performance compared to today’s constant pressure engines, producing up to 10$\%$ increased thrust. This new propulsion cycle will also reduce thruster size and/or weight, lower injection pressures, and are less susceptible to engine-damaging acoustic instabilities. Here we present a collective effort to benchmark performance and standardize operability of rotating detonation rocket engines to develop the RDRE technology readiness level towards a flight demonstration. Key detonation physics unique to RDREs, driving consistency and control of chamber dynamics across the engine operating envelope, are identified and addressed to drive down the variability and stochasticity observed in previous studies. This effort demonstrates an RDRE operating consistently across multiple facilities, validating this technology’s performance as the foundation of RDRE architecture for future aerospace applications.

## Introduction

There is an explosive growth in space missions for accelerating planetary exploration and commercial space activities. To achieve these missions, advanced rocket engine technologies are needed to make space access more reliable, routine, and cost-effective. Rocket engines are the most expensive component of launch vehicles, where only marginal engine performance improvements have been achieved in recent decades. Furthermore, the highest-performing propulsion cycles lead to extensive development costs and require high injection and chamber pressures, which necessitate large capacity propellant pumps that have cost and reliability concerns. Traditional engines are also susceptible to acoustic combustion instabilities, which typically are a costly part of the design and qualification process to ensure their suppression^1^. The rotating detonation rocket engine (RDRE) is an emerging engine technology that can revolutionize space propulsion. This detonation-based engine with supersonic combustion-driven shock waves offers several advantages over traditional rocket systems such as lower injection pressures^2^, a significant size/weight reduction due to the compact combustion zone^3,4^, lower pressure requirements for the gas generator and drive pumps^2^, less susceptibility to acoustic instabilities due to mode locking, and increased specific impulse performance on the order of 10$\%$^5,6^. Detonation-based rocket engine technology can create new cost-competitive markets that result in considerable savings and accelerate space exploration through versatility in engine thrust scaling and streamlined system integration.

The initial discovery of rotating detonation waves in rockets dates back to the early observations of spinning detonations in 1960 by Voitsekhovskii et al. with gaseous acetylene and oxygen^7^. Similarly, Nicholls et al. observed transverse combustion instabilities in rocket engines^8^ leading to the formation of rotating detonation waves in a rocket motor. Both investigations stemmed from programs seeking to suppress combustion instabilities in traditional rocket combustors^9^, a historical development challenge that has plagued high-performing rockets including the F-1 engine development for the Apollo program^10,11^. In the past decade, researchers have begun to investigate the potential of driving these instabilities to nonlinear saturation for engine performance purposes, bringing about the genesis of a novel detonation-based propulsion cycle for rockets. During this time, significant progress has been made in the research and development of rotating detonation engines^12–15^. A wide series of experimental investigations have explored various aspects of detonation-based propulsion systems including operability and performance^16–19^, injection response^4,20^, fundamental detonation structure^21–24^, effect of fuel and oxidizer compositions^25,26^, nozzle concepts^4,27^, system integration^28^, measurement techniques^29–31^ and flight technology^32,33^. Although there is a large body of work characterizing various aspects of rotating detonation waves, there remains to be a coordinated effort to unify the results gathered under perfectly matching conditions for RDRE verification and validation.

Detonation-based propulsion presents unique scientific challenges due to the complex combustion processes. Detonations create a shock-driven reacting flow field that is associated with large amounts of rapid energy transfer taking place across a vast range of time and length scales. Unlike conventional deflagration-based rocket engines, RDREs spontaneously excite operating modes that stabilize with one or more traveling detonation wave(s); the stability and strength of these waves directly affect combustion efficiency and engine performance. Stemming from the flame-acoustic coupling phenomenon of combustion instabilities^34^, detonation modes are governed by highly non-linear underlying physics including combined effects from chemical reaction kinetics, local inflow boundary conditions, and reactant mixture ratio. Stabilizing and controlling these operating modes are crucial towards developing this technology, as previous studies have shown that chamber wave dynamics have inherent large-scale sensitivities (e.g., inflow conditions) that can impact combustor operability and performance^16,35,36^. Therefore, to maximize the benefits of a detonation-based propulsion system, it is necessary to unify the experimental assessment for RDREs to reduce the stochasticity of the operating modes of these engines.

A coordinated research effort has been performed in this study to unify rotating detonation rocket propulsion. The experimental investigation of the same 1350 N thrust-class RDRE at four separate propulsion facilities is undertaken to benchmark the performance of the device and isolate the key mechanisms responsible for consistency amongst the detonation waves. The understanding gained from this experimental investigation is central towards developing the next generation RDRE flight demonstrator. Three operating conditions and a transition regime are studied, encompassing a range of varying performance levels and detonability within the engine operating space. Targeted two- to three-wave detonation modes are excited, demonstrating control for these conditions ranging from  60–75$\%$ of theoretical detonation wave speed and thrust levels of  350–625 N. Overall, providing well-defined inlet chamber boundary conditions through standardizing the flow metering systems, as well as unifying the measurement techniques for these highly transient devices, produced consistent results with minimal uncertainties for the chamber wave dynamics and performance amongst the different propulsion facilities. This sets a universal standard for future design studies and characterization that provides the necessary foundation to the burgeoning global efforts to develop the technology readiness level of RDREs.

## Results and discussion

### Rotating detonation rocket engine

**Figure 1:**
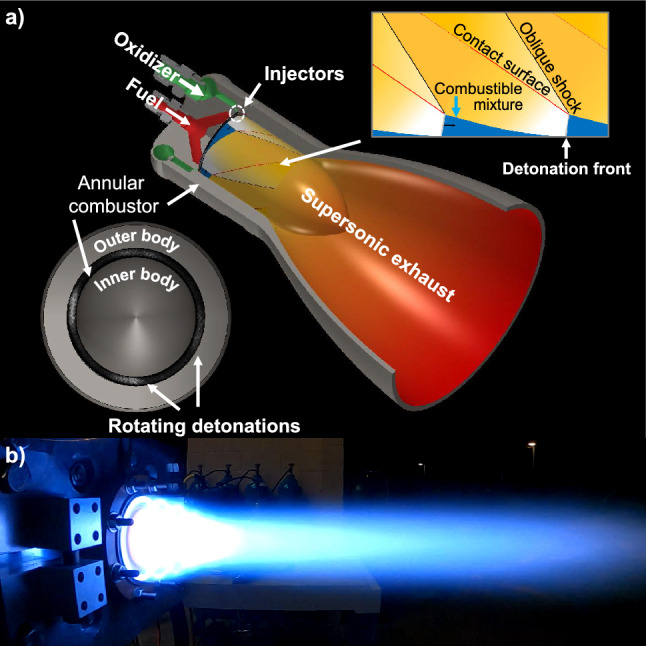
Views of a rotating detonation rocket engine including the (**a**) hardware schematic, and (**b**) engine firing of the canonical hardware.

A canonical rotating detonation rocket engine developed and used in this effort provides a scalable architecture that is capable of controlling high-strength detonation to generate thrust. The engine efficiently converts the chemical energy contained within the propellants (i.e., methane as the fuel and oxygen as the oxidizer) to kinetic energy through a detonation-based combustion process. Unique to RDREs, supersonic exhaust is directly created through the detonation process. To achieve efficient propellant mixing and detonation, the canonical RDRE incorporates a distinct annular configuration, with both an inner and outer body (see Fig. 1a) that geometrically promotes rotational operating modes due to flame-acoustic coupling^10^. The injection scheme is an unlike impinging doublet with heritage dating back to the F-1 rocket engine^11^, which is used to provide rapid propellant mixing to ensure reactant uniformity and produce robust detonation. Together, the annular chamber geometry and injector create a complex flow field with multiple stable rotating detonation(s) (see Fig. 1a) that power the engine. This new engine geometry (shown in operation in Fig. 1b) serves as part of the detonation-based rocket engine technology transition pathway directed by the United States Air and Space Force^37^.

**Figure 2:**
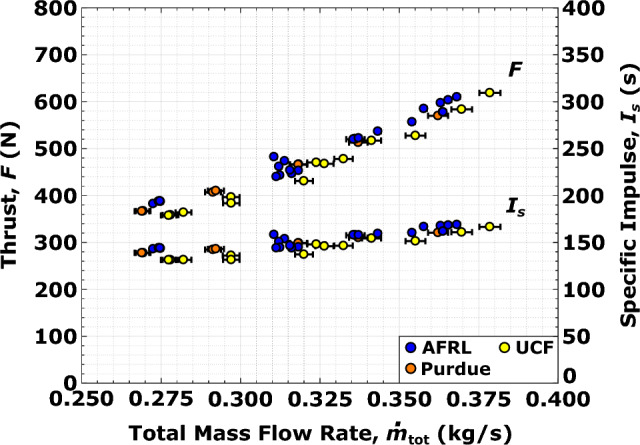
Engine performance summary showing (**a**) thrust *F* and (**b**) specific impulse $I_s$ as a function of total propellant mass flow rate $\dot{m}_\text{tot}$ at $\phi$ = 1.1. For certain tests, error bars are smaller than the symbols shown.

Engine thrust and specific impulse for the RDRE in the present study both monotonically map to total propellant flow rate as indicated in Fig. 2. The thrust produced ranges from 350 to 625 N with specific impulse ranging from 125 to 175 s for $\dot{m}_\text{tot}$ = 0.270–0.375 kg/s. Overall uncertainty on these engine performance metrics range from 0.5 to 1.5$\%$ and results show good agreement amongst all propulsion facilities; this confirms that consistent local inflow boundary conditions are critical for uniform detonation amongst all reported tests of this canonical RDRE.

**Figure 3:**
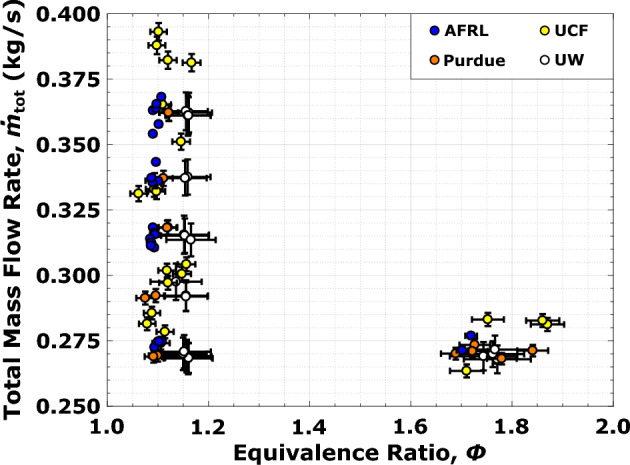
Flow condition test matrix for the canonical RDRE hardware tested at the four research facilities. For certain tests, error bars are smaller than the symbols shown.

Flow conditions are a function of equivalence ratio $\phi$ and total propellant mass flow rate $\dot{m}_\text{tot}$, which both influence detonation characteristics; $\phi$ controls reactant chemistry, while $\dot{m}_\text{tot}$ influences propellant fill recovery and initial detonation pressure. Fuel rich equivalence ratio conditions spanning $\phi$ = 1.1 to 1.7 have shown experimentally to achieve the highest performance for the gaseous propellant combination of methane ($\mathrm CH_4$) and oxygen ($\mathrm O_2$) in previous RDRE studies^4,17^, as well as provide thermodynamic benefits^5^ over conventional engines. Therefore, in order to explore sensitivities of this engine, two equivalence ratios at $\phi$ = 1.1 and 1.7 are considered at $\dot{m}_\text{tot}$ = 0.272 kg/s, along with total propellant mass flow rates ranging from 0.270 to 0.375 kg/s for a fixed $\phi$ = 1.1 (see Fig. 3); uncertainties in flow conditions range from 0.5 to 6$\%$ for equivalence ratio and 0.3–2.5$\%$ for total mass flow (see Table 1). Stable detonation at consistent operating modes is sustained across the entirety of these flow conditions at all propulsion facilities, proving for the first time that robust engine operation is achievable despite the stochasticity of underlying detonation physics.

**Table 1:** Measurement uncertainties in controlled experiment inputs.

Location	Ox. mass flow	Fuel mass flow	Total mass flow	Equivalence ratio
	(kg/s)	(kg/s)	(kg/s)	
AFRL	±8.65E−04 (0.3%)	±3.09E−04 (0.4%)	±9.19E−04 (0.3%)	±0.01 (0.5%)
Purdue	±29.5E−04 (1.0%)	±10.3E−04 (1.3%)	±31.3E−04 (0.9%)	±0.02 (1.7%)
UCF	±30.9E−04 (1.1%)	±8.55E−04 (1.1%)	±32.1E−04 (0.9%)	±0.02 (1.5%)
UW	±68.5E−04 (2.4%)	±23.1E−04 (2.8%)	±68.5E−04 (2.4%)	±0.07 (6.1%)

### Performance of the rotating detonation

**Figure 4:**
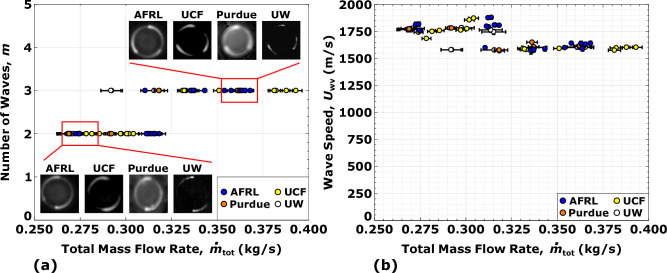
Summary of the detonation mode characteristics as a function of total propellant mass flow rate at $\phi$ = 1.1, depicting the (**a**) number of waves *m*, and (**b**) average wave speed $U_\text{wv}$. For certain tests, error bars are smaller than the symbols shown.

Unlike constant-pressure rocket combustors, detonation-based engines have unique operating modes that excite multiple detonation(s) traversing the annulus; the strength and number of waves influence engine performance, with peak thrust *F* and specific impulse $I_{s}$ for operating modes with the maximum detonative heat release at elevated pressure throughout the chamber^5^. For the investigated flow conditions, controlled operating modes with either two or three detonation waves are excited. The nominal flow condition ($\phi$ = 1.1, $\dot{m}_\text{tot}$ = 0.272 kg/s) has two waves, where the fuel rich ($\phi$ = 1.7, $\dot{m}_\text{tot}$ = 0.272 kg/s) and high flow rate ($\phi$ = 1.1, $\dot{m}_\text{tot}$ = 0.363 kg/s) conditions excite three waves. The mode transition between the nominal and high flow conditions, i.e., from two to three waves as shown in Fig. 4a, corresponds to wave speeds between 65-75$\%$ of the theoretical Chapman-Jouguet detonation speed ($D_\text{CJ}$ $\approx$ 2500 m/s for the nominal condition)^38^. Uncertainties in wave speed are under 1.5$\%$ of the values reported in Fig. 4b (see Table 2). In particular, this mode transition is consistently captured by all facilities in the region of $\dot{m}_\text{tot}$ = 0.300–0.325 kg/s. This transition demonstrates for fixed reactant chemistry that increasing the rate of reactant replenishment allows for more detonations to be supported. This is consistent with the wave stability criterion of Wolanski^39^, which provides an idealized approach to the minimum number of waves as

1 $$\begin{aligned} W = \frac{\dot{m}_{\text{tot}}}{\rho _{\text{tot}} l_{\text{cr}} w U_{\text{wv}}}, \end{aligned}$$

where $\rho _{\text{tot}}$ is the total propellant mixture density, $l_{\text{cr}}$ is the critical detonation reactant mixing zone height, *w* is the detonation channel width, and $U_\text{wv}$ is the wave speed. Therefore, when $\dot{m}_\text{tot}$ is increased for fixed $l_\text{cr}$, it will increase the overall number of waves within the channel.

**Table 2:** Uncertainties in measured wave characteristics and performance metrics.

Location	Op. frequency	Wave speed	Thrust	Specific impulse
	(kHz)	(m/s)	(N)	(s)
AFRL	±0.228 (1.1%)	±16.9 (1.1%)	±2.67 (0.5%)	±0.851 (0.5%)
Purdue	±0.101 (0.5%)	±7.50 (0.5%)	±5.10 (0.9%)	±2.17 (1.4%)
UCF	±0.087 (0.4%)	±8.18 (0.5%)	–	–
UW	±0.155 (0.7%)	±14.6 (0.9%)	–	–

**Figure 5:**
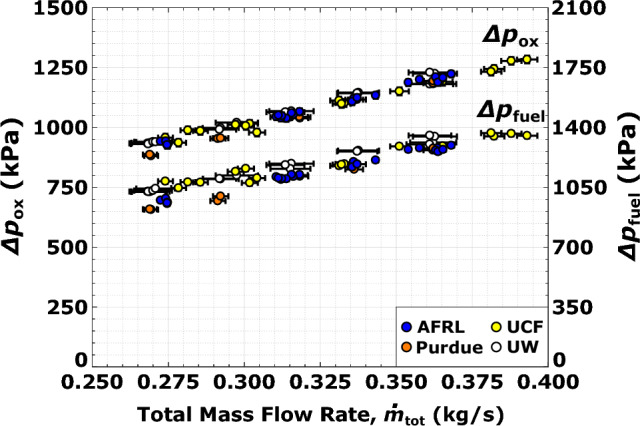
Injection pressure drop of the (**a**) oxidizer and (**b**) fuel as a function of total propellant mass flow rate at $\phi$ = 1.1.

Operation over a range of total propellant flow rate at fixed equivalence ratio is necessary in rocket applications where the ability to vary the thrust force is required (i.e., deep engine throttling), such as for space maneuvers and landing^40^. Due to the observed mode transition between the nominal ($\phi$ = 1.1, $\dot{m}_\text{tot}$ = 0.272 kg/s) and high flow rate ($\phi$ = 1.1, $\dot{m}_\text{tot}$ = 0.363 kg/s) conditions, emphasis is placed on confirming uniformity of the combustor operating conditions and propellant feed systems. Figure 5 shows a comparison of the pressure drops for the oxidizer and fuel injectors, as a function of total propellant mass flow rate at $\phi$ = 1.1. Data collected at the different propulsion facilities are in good agreement and exhibit the linear trend characteristic of metered compressible flow. The variation in measured injector pressure drop data, relative to this linear trend, is limited to ±45 kPa in the oxidizer plenums and ±90 kPa in the fuel plenums. Uncertainties in pressure drops range from 0.7 to 1.4$\%$ for the oxidizer plenums and 0.8–1.2$\%$ for the fuel plenums (see Table 3). Consistency in the propellant injector pressure drops between the test facilities is key because it indicates (1) inflow boundary conditions are comparable during testing, and (2) similar combustion chamber dynamics are excited.

**Table 3:** Uncertainties in measured system pressures.

Location	CTAPs	Ox. plenum	Fuel plenum	Ox. $\Delta P$	Fuel $\Delta P$
	(kPa)	(kPa)	(kPa)	(kPa)	(kPa)
AFRL	±9.24 (2.3%)	±10.3 (0.6%)	±10.4 (0.6%)	±13.9 (1.1%)	±14.0 (1.1%)
Purdue	±2.49 (0.6%)	±9.80 (0.6%)	±10.3 (0.6%)	±10.3 (0.9%)	±10.7 (0.8%)
UCF	±12.9 (3.1%)	±11.7 (0.7%)	±11.2 (0.6%)	±16.1 (1.4%)	±15.7 (1.2%)
UW	±5.28 (1.6%)	±7.34 (0.4%)	±9.18 (0.5%)	±9.00 (0.7%)	±10.6 (0.8%)

As part of this research effort, significant work is exerted to standardize consistent reactant zone conditions and eliminate the combustor operating mode stochasticity. Unlike conventional rockets, the propellant inflow boundary in RDREs interacts with the reaction zone due to the presence of traveling detonations. The detonation wave influences this boundary by increasing both the average chamber pressure and the size of a high-pressure zone trailing the wave. To counteract the interruption of propellant inflow due to these local high-pressure regions, plenum pressures increase to maintain the total propellant flow rate metered upstream of the combustor. Therefore, the pressure drop across the injector is a measure that captures both detonation-driven effects, and consistency between measured pressure drops at the same flow condition confirms the presence of similar propellant refresh processes at each facility.

Time-averaged chamber pressures are recorded using capillary tube attenuated pressure (CTAP) transducers, where the CTAP sensor locations closest to the injector face within the combustor are used in assessing the injector pressure drop. Propellant plenum pressures are recorded with conventional low-frequency transducers. Agreement in the recorded pressure drops between propulsion facilities across a wide range of total propellant flowrates and mixture ratios indicates that the established operating mode within the RDRE is characterized by similar strength detonation waves. A lack of apparent outliers in these pressure drops indicates the operation of rocket detonative combustors is not stochastic but is instead deterministic of the inflow and boundary conditions.

**Figure 6:**
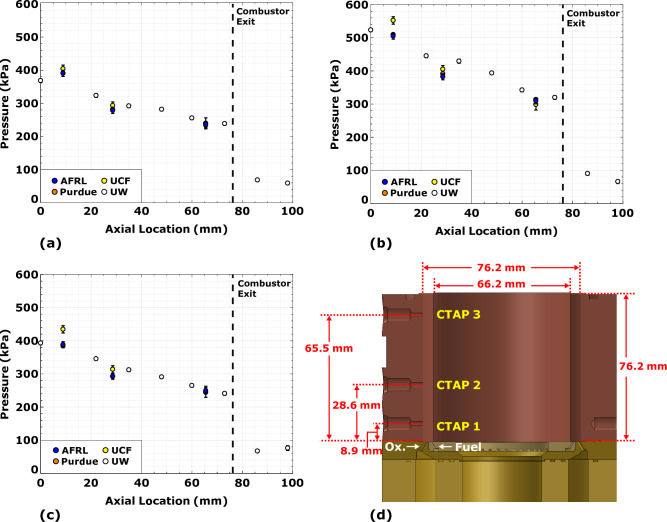
Axial chamber pressure profiles for (**a**) $\phi$ = 1.1 at $\dot{m}_\text{tot}$ = 0.272 kg/s, (**b**) $\phi$ = 1.1 at $\dot{m}_\text{tot}$ = 0.363 kg/s and (**c**) $\phi$ = 1.7 at $\dot{m}_\text{tot}$ = 0.272 kg/s, with (**d**) locations of CTAP pressure sensors indicated on schematic of laboratory scale rotating detonation rocket engine.

A CTAP measurement is made at various axial stations throughout the annular chamber. Facilities at the Air Force Research Laboratory (AFRL), Purdue University, and the University of Central Florida (UCF) record three static transducer measurements within the combustor, while the propulsion laboratory at the University of Washington (UW) records eight such measurements, two of which are downstream of the combustor exit. Axial pressure distributions for the three cases shown in Fig. 6 illustrate strong agreement between participants, and in all cases decreases as a function of axial location. Similarity in the shape of these pressure profiles indicates the heat addition from the traveling detonation wave occurs at comparable positions within the combustor; thus, standardization and control of the propellant flow metering between the propulsion facilities has eliminated variability in reported detonation wave characteristics.

A pressure decrease of more than 50$\%$ between the last internal and two external CTAP measurements collected by UW confirms the combustor is operating under a choked condition for each of the three test conditions in Fig. 6. The combination of heat addition from the detonation and total propellant mass flow rate is sufficient to produce a sonic plane, effectively eliminating the need for the converging section of a nozzle. A complete summary of the experimental parameters collected within this study for all propulsion facilities can be found in the Supplementary Material.

## Materials and methods for standardization

### Engine description

The rotating detonation rocket engine was developed using the design guidelines of Bykovskii et al.^35^, which features a 76.2 mm outer diameter annulus that is 76.2 mm long and 5 mm wide (see Fig. 6). The gaseous propellants, ultra-high purity methane and oxygen, flow into the chamber through 72 unlike flat impinging injector element pairs evenly spaced azimuthally around the annulus. Both the fuel and oxidizer injection orifices are inclined 30 degrees from the axial centerline, with the fuel injection orifices placed towards the inner diameter. Each element is designed to impinge at a height of 2.16 mm from the injector face at the centerline of the 5 mm annulus. The fuel and oxidizer injection orifices have diameters of 0.787 and 1.245 mm, respectively. These diameters are sized for an injector-to-annulus area ratio ($A_\text{inj}$/$A_\text{annul}$) of 0.110. This allows the fuel and oxidizer manifold pressures to be high enough for choked flow across the injector, with the exception of during a high-pressure wave passage event over an injection site.

All propellant flow rates are similarly metered under this effort using critical flow venturi nozzles (CFVNs), defined by the American Society of Mechanical Engineers (ASME) standard^41^. This flow metering approach operates using choked flow through the venturi, where flow at the throat propagates at the local sound speed of the working fluid and isolates upstream flow from downstream perturbations. This allows the flow to become solely a function of pressure upstream of the venturi and insensitive to pressure fluctuations downstream of the metering device, as long as choked flow persists. This is crucial for accurate flow metering in oscillatory devices such as RDREs that inherently have large-scale spatiotemporal pressure fluctuations present within the device due to the traveling detonation(s).

For ignition, the pre-detonator tube operates using $\mathrm CH_4$ and $\mathrm O_2$, and fires tangentially into the chamber axially located close to the injection plane. This configuration produces a planar detonation in the tube through a deflagration-to-detonation (DDT) transition event^42^, upon ignition of the premixed mixture within an upstream reservoir (vol. 53 $\mathrm cm^3$).

Chamber pressure is measured using CTAP static sensors, axially located 8.9, 29.2 and 65.5 mm downstream of the injection plane in the RDRE geometry studied by AFRL, Purdue, and UCF. The five CTAP locations in the UW geometry variant are 22.2, 34.9, 47.6, 60.3, and 73.0 mm downstream of the injector plane. CTAP sensors provide a temporally averaged measurement from the oscillatory pressure field by implementing a passive acoustic filter, i.e., stand-off tube, with the sensor located at the end. The *l*/*d* ratio of this standoff tube is based on the work of Stevens et al.^43^, ranging from approximately 650 - 1325 for AFRL, Purdue and UCF, and 145 for UW. Additionally, two high-frequency pressure transducers (e.g., PCB model 113B24, 111A24) are installed within the fuel and oxidizer plenums to characterize the acoustic field directly upstream of the injector plate. Finally, engine thrust is measured using a horizontal thrust stand with a 1100 N load cell.

In addition to the other measurements, the imaging approach used to characterize the operating mode is standardized across the four research facilities. High-speed visible imaging into the annulus at a minimum of 150 kfps at a resolution of 256x256 pixels is used to observe and capture the traveling detonation waves. The camera (e.g., Phantom v2512, Photron SA1.1) is positioned downstream of the engine within a protective enclosure with an optical window and is focused on the injection plane, either in a direct line-of-sight configuration (AFRL, UW) or off-axis reflection using a mirror (Purdue, UCF). Comparable long focal length lenses are implemented to resolve the RDRE annulus in the field of view and ultimately provide low uncertainties on detonation wave speed.

### Steady test measurements

During steady combustor operation, the propellant injector pressure drops are recorded. The pressure drop $\Delta p_\text{prop}$ is evaluated as the change in pressure between the propellant plenum $p_\text{plen,prop}$ and combustion chamber $p_\text{comb}$ (first sidewall CTAP data), and is given by

2 $$\begin{aligned} \Delta p_\text{prop} = p_\text{plen,prop} - p_\text{comb}. \end{aligned}$$

Propellant flow is metered upstream of the combustor by a pair of CFVNs as described in the Uncertainty Analysis section. The measured plenum pressures reflect the required pressure to ensure continuity with these CFVNs, the injector blockage due to detonation transit, and the injector discharge coefficient. Combustion chamber pressure is obtained from CTAP static transducer measurements located axially near the injector. In AFRL, Purdue, and UCF measurements, this transducer is located 8.9 mm from the injector face and in UW measurements, this transducer is installed flush (0 mm) with the injector face.

**Figure 7:**
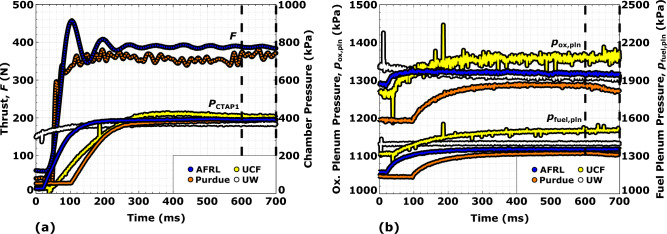
Transient run data for the $\phi$ = 1.1, $\dot{m}_\text{tot}$ = 0.272 kg/s (nominal) condition showing (**a**) thrust, (**b**) CTAP1 sensor chamber pressure, (**c**) oxidizer and (**d**) fuel plenum pressures.

The transient operation of the RDRE combustor at a total propellant flow rate of 0.272 kg/s and an equivalence ratio of 1.1 is presented in Fig. 7. A standard test consists of 500-750 ms of RDRE combustor operation following ignition, with data from the final 100 ms of this period used to report steady test parameters as denoted by the dashed lines in Fig. 7. Propellants flow through the combustor prior to the 500–750 ms test window to ensure the flow metering systems and propellant plenums are properly pressurized and have reached steady-state conditions. While the duration of this pre-ignition propellant flow is facility dependent, the transient data in Fig. 7 is aligned such that the final 100 ms of combustor operation occurs between 600 and 700 ms.

Thrust measurements from AFRL and Purdue show good agreement at 387±2.22 N and 367±5.10 N, respectively. Oscillations present in the thrust measurements immediately following ignition are the result of the impulsive load exciting the natural frequency of the thrust stands. The largest components of these oscillations damp out in 50–200 ms, long before the data collection window at the end of the test. From these measurements, it is apparent that the thrust produced by the RDRE is both steady and quick to stabilize.

In contrast, the transient pressure measurements within the chamber (Fig. 7a), and propellant plenums (Fig. 7b) are slower to respond. The chamber pressure, as measured by CTAP sensors, require over 100 ms to react to the sudden increase in combustor pressure from the detonation wave. As the name suggests, the 0.9–1.8 m long CTAP tubes at the four facilities attenuate the higher frequency components of the step increase in combustion chamber operating pressure, but yield steady values in the final 100 ms of the test. Data from these measurements are in good agreement.

The oxidizer and fuel plenum pressures also require several hundred milliseconds to reach a new steady condition (see Fig. 7b). Where the CTAP transducer measurements are typically low prior to ignition due to measuring the static pressure of a non-reacting flow, the plenum pressures prior to combustor ignition are over 1000 kPa and represent the required pressure to ensure mass continuity between the propellant injector and the flow metering CFVNs. Increase in the plenum pressures upon ignition is a result of the partial injector blockage due to the high-pressure region trailing the traveling detonation wave. The rate of this change is dependent on the volume between the flow metering CFVN and propellant injector and is facility dependent. As with the thrust and combustion chamber CTAP measurements, the 500-750 ms combustor test time is sufficient to ensure this measurement stabilizes prior to the 100 ms of data collection.

### Image processing

To standardize the processing of the high-speed images, the extraction of modal properties is accomplished using the procedure developed by Bennewitz et al.^44^. Each group utilizes the same back-end imaging methodology with only slight variations. UCF uses a different approach in finding cells to locate the annulus due to the use of a mirror downstream of the exhaust. During high mass flow rate conditions, the RDRE exhaust can cause the mirror to wobble, resulting in fluctuations in annulus location within the standard 1000 frame interval. To mitigate this fluctuation, the top cells within 20% of the maximum pixel intensity in each frame is used for the Taubin circle fit, producing an individual circle fit for each frame. This process maintains the same level of accuracy in tracking the annulus but is associated with a significant increase in computational cost. While AFRL and Purdue image at 200 kfps, UCF and UW image at 150 kfps and 240 kfps, respectively; in all cases the number of images in a frame set was controlled to reduce the uncertainty in average wave speed measurement. For example, most groups bin their back-end imaging into 1000 frame intervals, corresponding to the same time intervals for each image set. However, UCF analyzes 2000 frame sets with a 50% overlap. With this change, the 1000 frame increment is still maintained but the uncertainty is decreased by approximately 50%, bringing it in line with reported values for other groups.

Although each group derives their image processing algorithm from the process shown by Bennewitz et al.^44^, synthetic data are created to ensure that each independently developed algorithm produces equivalent results. The synthetic data are produced with a set of prescribed wave characteristics, as shown in Table 4. These data are then processed by the algorithms created by each group, confirming that equivalent results are achieved.

**Table 4:** Prescribed wave properties of synthetic data used for consistency confirmation.

Interval	1	2	3	4	5	6
Clockwise (CW)						
Num. of Wvs.	3	3	3	3	–	–
Wv. Spd. (m/s)	2383	2234	2085	2085	–	–
Op. Freq. (kHz)	32	30	28	28	–	–
Counterclockwise (CCW)						
Num. of Wvs.	–	4	4	4	4	4
Wv. Spd. (m/s)	–	1955	2234	2234	2122	2011
Op. Freq. (kHz)	–	35	40	40	38	36

### Uncertainty analysis

As part of standardizing the data processing approach, a detailed uncertainty analysis is performed for the collected experimental data, providing a valuable assessment of the measurement error bands. Two sources of uncertainty are considered including Type A and B, where Type A uncertainties are based on frequency distributions taken from a measured quantity sample and are evaluated with a statistical analysis of each measurement series^45^. The Type A uncertainty, i.e., an uncertainty associated with fluctuations (standard deviations) around the mean, $dx_{TypeA}$ is evaluated using

3 $$\begin{aligned} dx_\text{TypeA} = z_{\alpha /2}\frac{s_x}{\sqrt{N}}, \end{aligned}$$

where $s_x$ is the sample standard deviation, $\alpha$ is the significance level, and *N* is the sample size. The sample standard deviation determines the variability of the measured values around the mean or expected value. The significance level defines the confidence interval, which describes the range of values that is likely to contain the true value. For the uncertainties presented in this analysis, $\alpha$ is chosen to be 0.05, corresponding to a 95% confidence. Since the sample size is sufficiently large, the data sets are considered to have normal distributions about a mean value, which prescribe the value of $z_{\alpha /2}$ as 1.96.

Type B uncertainties are evaluated based on a variable-specific accuracy evaluation^45^. This form of uncertainty arises from factors such as the manufacturer-specified tolerances, calibration accuracy, manufacturing variances, and uncertainties assigned to reference data taken from handbooks, and are independent from the statistical analysis of the measurements. The following section describes the Type B uncertainty calculation methodology for each major measurement parameter of interest and provides a summary of the uncertainties reported by each propulsion facility.

Pressure measurement uncertainty (*dp*) was calculated with

4 $$\begin{aligned} dp = \sqrt{(C_1dV)^2+(VdC_1)^2+(dC_0)^2+(dp_\text{atm})^2+2V_\text{cov}(C_0,C_1)}, \end{aligned}$$

using the method described by Lightfoot et al.^46^. Table 5 describes the various components considered in this analysis.

**Table 5:** Description of pressure uncertainty components.

Quantity	Variable	Description
Voltage	*V*	Transducer voltage signal
Voltage uncertainty	*dV*	Uncertainty in volt. sign. derived from sign. fluct. and trans. full-scale range
Calibration coefficients	$C_1, C_0$	Transducer calibration curve fit coefficients
Coefficient uncertainties	$dC_1, dC_0$	Uncerts. in coeff. derived from supply pressure, voltage, and data spread
Atmospheric pressure uncert.	$dp_\text{atm}$	Uncertainty in transducer from manufacturer specifications

The primary source of pressure measurement uncertainty arises from the calibration coefficients $C_1$ and $C_0$. The calibration coefficients, the respective uncertainties of the calibration coefficients, and the covariance between the two coefficients are calculated using a weighted least-squares linear regression method specified by Mathioulakis et al.^47^. Uncertainty variation amongst the propulsion facilities is primarily due to differing transducer ranges. Uncertainties in pressure differences are calculated from the individual pressure measurement uncertainties, as shown in

5 $$\begin{aligned} d\Delta p_\text{ox} = \Delta p_\text{ox}\sqrt{\left( \frac{dp_\text{ox}}{\Delta p_\text{ox}}\right) ^2+\left( \frac{dp_\text{CTAP1}}{\Delta p_\text{ox}}\right) ^2}, \end{aligned}$$

and

6 $$\begin{aligned} d\Delta p_\text{fuel} = \Delta p_\text{fuel}\sqrt{\left( \frac{dp_\text{fuel}}{\Delta p_\text{fuel}}\right) ^2+\left( \frac{dp_\text{CTAP1}}{\Delta p_\text{fuel}}\right) ^2}. \end{aligned}$$

Uncertainty associated with propellant mass flow rate through a CFVN, $\dot{m}_\text{vent}$, is calculated using

7 $$\begin{aligned} \dot{m}_\text{vent} = \frac{A_t p_0 C_d C_\text{FF}}{\sqrt{RT_0}}, \end{aligned}$$

where $A_t$ is the CFVN throat area, $p_0$ is the propellant total pressure, $T_0$ is the propellant total temperature, *R* is the specific gas constant, $C_d$ is the CFVN discharge coefficient, and $C_{FF}$ is the critical flow function. Both the specific gas constant and the critical flow function are evaluated for each propellant using the NIST REFPROP database^48^.

The uncertainty in measured mass flow rates ($d\dot{m}_i$) is calculated using the approach from Lightfoot et al.^46^ and is written as

8 $$\begin{aligned} \begin{aligned} d\dot{m}_i = \dot{m}_i\sqrt{\left( \frac{dA_t}{A_t}\right) ^2+\left( \frac{dC_d}{C_d}\right) ^2+\left( \frac{dC_{FF_i}}{C_{FF_i}}\right) ^2+...} \\ \overline{\left( \frac{dp_i}{p_i}\right) ^2 +\left( \frac{dR}{2R}\right) ^2+\left( \frac{dT}{2T}\right) ^2} \end{aligned} \end{aligned}$$

To quantify $d\dot{m}_i$, the pressure measurement uncertainty is calculated using Eq. 4. In addition, temperature measurement uncertainty is determined using a similar method, considering the calibration coefficients and manufacturer accuracy specifications. Uncertainties associated with gas properties are assumed to be negligible, as the value for the universal gas constant has been established in previous literature and is taken as 8.314 J/mol-K per the CODATA database values^49^. Using the uncertainties associated with each reactant flowrate, the calculated uncertainty in total mass flow rate and equivalence ratio are evaluated using

9 $$\begin{aligned} d\dot{m}_\text{tot} = \dot{m}_{tot}\sqrt{\left( \frac{d\dot{m}_\text{ox}}{\dot{m}_\text{tot}}\right) ^2+\left( \frac{d\dot{m}_\text{fuel}}{\dot{m}_\text{tot}}\right) ^2}, \end{aligned}$$

and

10 $$\begin{aligned} d\phi = \phi \sqrt{\left( \frac{d\dot{m}_\text{ox}}{\dot{m}_\text{ox}}\right) ^2+\left( \frac{d\dot{m}_\text{fuel}}{\dot{m}_\text{fuel}}\right) ^2}, \end{aligned}$$

respectively.

The main contributions to mass flow rate measurement uncertainty are the sonic nozzle discharge coefficient and the measured gas temperature.

The Type B uncertainties associated with measured operational frequency and wave speed are evaluated considering the factors of spatial resolution and sample size, based on

11 $$\begin{aligned} df_\mathrm{det_B}=\frac{f_s}{2N}, \end{aligned}$$

and

12 $$\begin{aligned} dU_\mathrm{dwv_B}=\frac{f_s\pi d}{2Nm}, \end{aligned}$$

where $f_s$ is the high-speed camera frame rate, *N* is the number of frames in a given analysis bin, *d* is the annulus center diameter, and *m* is the number of waves observed during the test. The total uncertainties in operational frequency and wave speed can be calculated once the individual uncertainty components have been evaluated, using

13 $$\begin{aligned} df_\text{det}=\sqrt{(df_\mathrm{det_A})^2+(df_\mathrm{det_B})^2}, \end{aligned}$$

and

14 $$\begin{aligned} dU_\text{wv}=\sqrt{(dU_\mathrm{wv_A})^2+(dU_\mathrm{wv_B})^2}, \end{aligned}$$

where $df_{det_A}$ and $dU_{wv_A}$ are the Type A uncertainty contributions to operational frequency and wave speed, respectively.

Thrust measurement uncertainty is calculated similarly to that of pressure, using

15 $$\begin{aligned} dF = \sqrt{(C_1dV)^2+(VdC_1)^2+(dC_0)^2+2V_\text{cov}(C_0,C_1)}. \end{aligned}$$

Again, the calibration coefficients $C_0$ and $C_1$ contribute the most error to the total thrust uncertainty calculation. Finally, uncertainty in specific impulse is derived from computed uncertainties in thrust and total mass flow rate, as given by

16 $$\begin{aligned} dI_s = I_s\sqrt{\left( \frac{dF}{F}\right) ^2+\left( \frac{d\dot{m}_\text{tot}}{\dot{m}_\text{tot}}\right) ^2}. \end{aligned}$$

## Concluding remarks

Rotating detonation rocket engines offer the potential to revolutionize space missions and exploration. This new detonation-based propulsion engine will allow the delivery of larger payloads to space through a more efficient and compact launch system and extend in-space vehicle life, as this engine technology produces increased performance, uses lower injection pressures, and is less susceptible to acoustic instabilities. Rotating detonation waves, i.e., supersonic combustion-driven shocks, generate significant heat release at intense pressures and temperatures, allowing more useful available work to be extracted from combustion. As a foundational step to advancing detonation-based engine technology, a collective effort has been undertaken by four propulsion facilities to standardize operability and benchmark performance of rotating detonation rocket engines to revolutionize future rocket propulsion. By standardizing the rocket engine facilities, the same two- to three-wave stable operating modes are observed by each group for propellant flow rates ranging from 0.270 to 0.375 kg/s at a fixed equivalence ratio of 1.1. Within this flow regime, a mode transition is successfully captured between $\dot{m}_\text{tot}$ = 0.300–0.325 kg/s, and is supported by RDRE wave stability theory. Agreement amongst the local operating mode produces consistent engine performance across the investigated flow conditions, ranging from *F* = 350–625 N and $I_s$ = 123–175 s for the data collected at the various propulsion facilities. To produce a cohesive validation set of experimental results for this canonical RDRE, measurement standards including flow metering, high-speed image capturing and performance measurements are developed, in addition to data reduction approaches including high-speed image processing and uncertainty quantification. By providing detailed understanding towards stabilizing these operating modes for RDREs under this collective effort, this work serves as a path towards future design optimization studies to bolster the unique benefits of a detonation-based propulsion cycle for future space missions.

### Supplementary Information

Supplementary Tables.

## Data Availability

The datasets used and/or analysed during the current study available from the corresponding author on reasonable request.
