# Supplementary material for: Experimental validation of rotating detonation for rocket propulsion
Source: Sci Rep. 2023 Aug 30;13:14204. doi: 10.1038/s41598-023-40156-y (PMC10469202; doi:10.1038/s41598-023-40156-y)
Supplement: Supplementary file 1 — Supplementary Tables. [file 41598_2023_40156_MOESM1_ESM.pdf]

Appendix A Test Condition Summary Data

Table A1 Experimental Condition Summary: Engine Performance.

Cond.	Loc.	Equiv. Ratio, ϕ	Tot. Mass Flow Rate, $\dot{m}_{\text{tot}}$ (kg/s)	Thrust, F (N)	Spec. Impulse I_s (s)	CTAP_1 (kPa)	CTAP_2 (kPa)	CTAP_3 (kPa)
$\dot{m}_{\text{tot}} = 0.272$ kg/s	AFRL	1.11±5.09E-03	0.275±7.06E-04	387±2.22	144±0.902	390±9.21	279±9.22	236±9.22
	Purdue	1.10±17.9E-03	0.269±22.9E-04	367±5.10	139±2.39	391±2.45	282±1.83	234±1.56
$\phi = 1.10$	UCF	1.11±17.1E-03	0.274±24.4E-04	-	-	404±11.1	293±11.1	240±16.6
	UW	1.15±46.5E-03	0.270±61.9E-04	-	-	350±5.22	308±5.04	248±4.83
$\dot{m}_{\text{tot}} = 0.363$ kg/s	AFRL	1.10±5.46E-03	0.364±9.19E-04	578±2.66	162±0.851	505±9.29	382±9.22	310±9.22
	Purdue	1.12±18.6E-03	0.362±31.3E-04	570±5.10	160±2.17	509±3.10	391±2.41	313±1.98
$\phi = 1.10$	UCF	1.11±16.9E-03	0.365±32.1E-04	-	-	552±11.1	405±11.1	298±16.6
	UW	1.16±43.3E-03	0.363±72.3E-04	-	-	492±5.26	437±5.71	333±5.37
$\dot{m}_{\text{tot}} = 0.272$ kg/s	AFRL	1.70±11.5E-03	0.271±10.9E-04	394±2.23	148±1.03	388±9.22	293±9.22	249±9.22
	Purdue	1.69±29.5E-03	0.270±22.6E-04	368±5.10	139±2.36	385±2.37	292±1.87	244±1.63
$\phi = 1.70$	UCF	1.75±31.4E-03	0.283±24.9E-04	-	-	435±11.1	314±11.1	246±16.6
	UW	1.77±71.4E-03	0.272±53.6E-04	-	-	374±4.67	329±4.57	255±4.85

Table A2 Experimental Condition Summary: Detonation Mode Parameters.

Cond.	Loc.	Equiv. Ratio, ϕ	Tot. Mass Flow Rate, $\dot{m}_{\mathrm{tot}}$ (kg/s)	$p_{\mathrm{ox,pln}}$ (kPa)	$p_{\mathrm{fuel,pln}}$ (kPa)	Num. Waves, m	Wave Spd., U_{wv} (m/s)	Oper. Freq., f_{det} (kHz)
$\dot{m}_{\mathrm{tot}} = 0.272$ kg/s	AFRL	1.11±5.09E-03	0.275±7.06E-04	1316±10.3	1352±10.3	2	1754±14.2	15.7±12.7E-02
	Purdue	1.10±17.9E-03	0.269±22.9E-04	1272±7.45	1311±7.45	2	1776±11.3	15.9±10.1E-02
$\phi = 1.10$	UCF	1.11±17.1E-03	0.274±24.4E-04	1361±11.2	1489±11.2	2	1743±7.85	15.6±7.78E-02
	UW	1.15±46.5E-03	0.270±61.9E-04	1308±6.63	1399±6.63	2	1768±21.6	15.8±15.1E-02
$\dot{m}_{\mathrm{tot}} = 0.363$ kg/s	AFRL	1.10±5.46E-03	0.364±9.19E-04	1713±10.3	1784±10.3	3	1593±16.9	21.4±22.8E-02
	Purdue	1.12±18.6E-03	0.362±31.3E-04	1743±11.7	1787±9.80	3	1618±7.50	21.7±10.1E-02
$\phi = 1.10$	UCF	1.11±16.9E-03	0.365±32.1E-04	1743±11.7	1784±10.3	3	1604±8.18	21.5±8.69E-02
	UW	1.16±43.3E-03	0.363±72.3E-04	1748±7.35	1870±7.35	3	1606±14.6	21.5±15.5E-02
$\dot{m}_{\mathrm{tot}} = 0.272$ kg/s	AFRL	1.70±11.5E-03	0.271±10.9E-04	1192±10.3	1756±10.3	3	1458±9.82	19.6±13.2E-02
	Purdue	1.69±29.5E-03	0.270±22.6E-04	1132±6.68	1685±6.68	3	1456±7.47	19.6±10.0E-02
$\phi = 1.70$	UCF	1.75±31.4E-03	0.283±24.9E-04	1195±11.3	1912±11.3	3	1431±8.00	19.2±8.38E-02
	UW	1.77±71.4E-03	0.272±53.6E-04	1150±6.47	1787±6.47	3	1472±15.4	19.7±16.9E-02
